# First report of *Meloidogyne hapla* on kiwifruit in South Africa

**DOI:** 10.21307/jofnem-2020-082

**Published:** 2020-08-18

**Authors:** Ebrahim Shokoohi, Phatu W. Mashela

**Affiliations:** Green Biotechnologies Research Centre of Excellence, University of Limpopo, Private Bag X1106, Sovenga 0727, South Africa

**Keywords:** *Actinidia* spp., Limpopo province, *Meloidogyne* species, Molecular phylogeny, Temperate fruit crop

## Abstract

Kiwi is becoming one of the most important fruit in subtropical regions of South Africa with altitudes that confer sufficient chilling requirements. During a survey on biodiversity of plant-parasitic nematodes of kiwi in Magoebaskloof in Limpopo Province, several plant-parasitic nematodes were discovered, with *Meloidogyne* species occurring at the highest frequency. Nematodes were sampled from roots and the rhizosphere of one stunted Kiwi tree, extracted using the tray method and then fixed. The morphological characters fit well with those of *M. hapla*. The molecular approach using ITS and 28S rDNA, along with the related phylogenetic analysis, placed the examined population in a group with other populations of *M. hapla.* Kiwi is being reported as a new host for *M. hapla* in South Africa.

Worldwide, the kiwi (*Actinidia* spp.) fruit trees are increasingly being cultivated in temperate regions. The genus *Actinidia* comprises more than 70 species (Peng et al., 2019), with *A. deliciosa* being the most popular across the world. At Magoebaskloof, Limpopo Province, South Africa, kiwifruits are produced in subtropical regions much closer to the tropical regions in the Southern hemisphere. The location has a high altitude that confers temperate climatic conditions, which are suitable for the production of kiwifruits. Subsequently, the kiwifruit is becoming increasingly important outside of temperate regions in South Africa.

During November 2019, root samples were collected from roots of kiwifruit trees in the Magoebaskloof area (S: 23°52′43′′; E: 29°56′13′′) (Fig. 1). Roots were washed, cut into pieces and mature female specimens were removed using a scalpel, using a Zeiss stereomicroscope. The specimens were fixed with a hot 4% formaldehyde solution and transferred to anhydrous glycerin using De Grisse’s (1969) method. The characteristics perineal patterns of the second-stage juvenile (J2) were used to identify the test *Meloidogyne* species (Perry et al., 2009).

**Figure 1:**
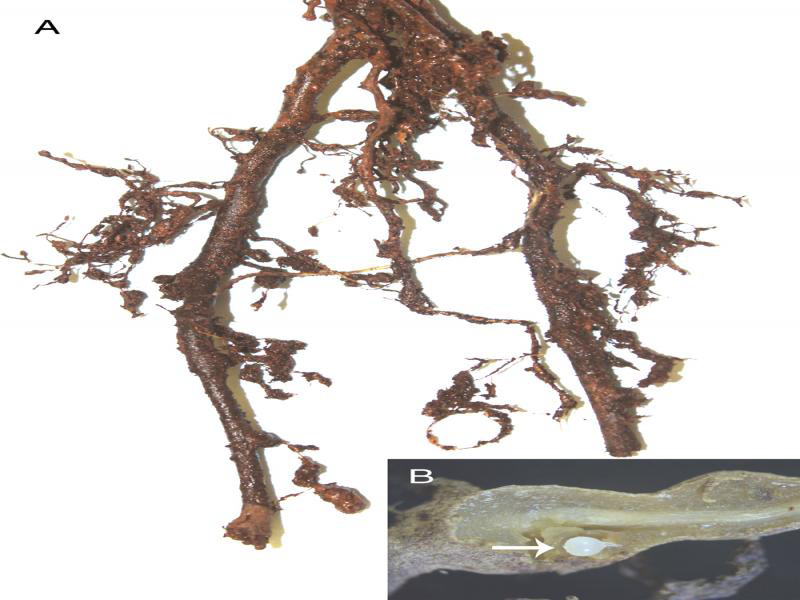
*Meloidogyne hapla* Chitwood, 1949. (A) Roots of kiwi tree affected. (B) Mature female on the root.

The molecular characterization followed the methods described in Álvarez-Ortega et al. (2019). The ribosomal ITS and LSU sequences were analyzed and aligned using the program BioEdit (Hall, 1999), aligned using CLUSTAL W (Thompson et al., 1994). The length of each alignment was 946 and 1186 bp for ITS rDNA and 28S rDNA, respectively. Bayesian inference was used to reconstruct the phylogeny, with Bayesian trees generated using the Bayesian inference method as implemented in the program MrBayes 3.1.2 (Ronquist and Huelsenbeck, 2003). The GTR + I + G model was selected using jModeltest 2.1.10 (Guindon and Gascuel, 2003; Darriba et al., 2012). Analysis using the GTR + I + G model was initiated with a random starting tree and ran with the Markov chain Monte Carlo (MCMC) for 10^6^ generations for ITS and 28S rDNA. The trees were visualized with the TreeView program. The original partial ITS rDNA and 28S (D2-D3 expansion) sequence of *M. hapla* were deposited in GenBank under the accession numbers MT256109 and MT258534, respectively. The morphological and molecular analyses confirmed that the species was *M. hapla*.

Morphometric mean, standard deviation and range values of *M. hapla* J2 were (*n* = 10): *L* = 337 ± 15.5 (322-353) μm; *a* = 30.8 ± 2.4 (28-32); *b* = 3.7 (*n* = 1); *c* = 8.5 ± 1.0 (7.4-9.5); stylet length = 12.6 ± 0.6 (11.9-13.0) μm; center of the median bulb to anterior end = 48.8 ± 2.6 (46-51) μm; excretory pore to anterior end = 67 ± 1.0 (66-68) μm; length body to the middle of genital primordium = 208.3 ± 8.6 (199-216) µm, hyaline part of tail length = 11.2 ± 1.3 (10.0-12.6) μm and tail length = 39.6 ± 3.1 (37-43) μm. The J2 had the smooth and spherical head, with a tail tapering to a blunt or rounded terminus. This species already has been studied from tropical areas of Africa (Whitehead, 1969), India (Waliullah, 2005), Chile (Carneiro et al., 2007), Brazil (Somavilla et al., 2011), Italy (D’Errico and Giacometti, 2012), and Turkey (Akyazi et al., 2017). Configuration of perineal patterns of females, their morphologies and morphometrics of *M. hapla* J2 were similar to those reported previously for isolates of this nematode species from tropical areas of Africa (Whitehead, 1969). However, the stylets of J2 from South Africa were slightly longer than those of the studied by Whitehead (1969). In comparison with Turkish population of *M. hapla*, they differ in body length (322-353 vs 340-440 µm), excretory pore to anterior end (66-68 vs 60.7-82.4 µm), hyaline part of the tail (10-12.6 vs 12-18.5 µm), and tail length (37-43 vs 50.2-54.8 µm). Although, our population of *M. hapla* showed no significant differences with those second-stage juveniles of *M. hapla* studied by Handoo et al. (2005).

The sequence lengths flanked by the forward primer TW81 [5′-GTTTCCGTAGGT GAACCTGC-3′] and AB28 [5′-ATATGCTTAAGTTCA GCGGGT-3′] (Joyce et al., 1994); D2A (5″-ACAAGTACCGTGAGGGAAAGTTG-3″) and the reverse primer D3B (5″-TCGGAAGGAACCAGCTACTA-3″) (De Ley et al., 1999) of the ITS rDNA and 28S region of *M. hapla* isolate 505 and 702 base pairs long, respectively. The nBlast test of ITS rDNA showed that the test population had only one base pair, which was different to those of *M. hapla* from South Korea (MK188473), Japan (LC030357), and Taiwan (KJ572385), all with 99% similarity. Despite high similarity (99%) with *M. hapla* populations, our sequence of *M. hapla* showed the lowest similarity, 85% with *M. incognita* (KJ739707) and *M. javanica* (KJ739709), and 79% with *M. enterolobii* (KM046989) using ITS rDNA marker. The nBlast of 28S rDNA showed four bp differences with 98% similarity with the Chinese (MN752204; KJ755183) and Ethiopian population (KP410845). Despite high similarity (98%) with *M. hapla* populations, our sequence of *M. hapla* showed the lowest similarity, 89% with *M. incognita* (JX100425), *M. javanica* (JX100426), and *M. enterolobii* (KJ146862) using 28rDNA marker. Therefore, molecular result confirmed our populations as *M. hapla*.

The phylogenetic analysis using ITS and 28S rDNA, placed the South African *M. hapla* population in a clade together with other *M. hapla* populations (Figs. 2, 3). The molecular characterization of several species of *M. hapla* suggested that they formed a monophyletic group. Findings in the current study were in agreement with the phylogenies of *Meloidogyne* species studied using 18S rDNA, ITS, 28S rDNA and COII of mtDNA (De Ley et al., 2002; Tigano et al., 2005; Tao et al., 2017). Two permanent microscope slides containing the perennial patterns and female and J2 of *M. hapla* were deposited in the Nematology collection of the University of Limpopo, South Africa. According to literature, this is the first record of *M. hapla* from kiwifruits in South Africa. Besides, ITS and 28S rDNA information of this species are being reported for the first time. *M. hapla* was associated with kiwifruits in Limpopo Province and therefore, host-status studies are necessary to find out the severity of this root-knot nematode.

**Figure 2:**
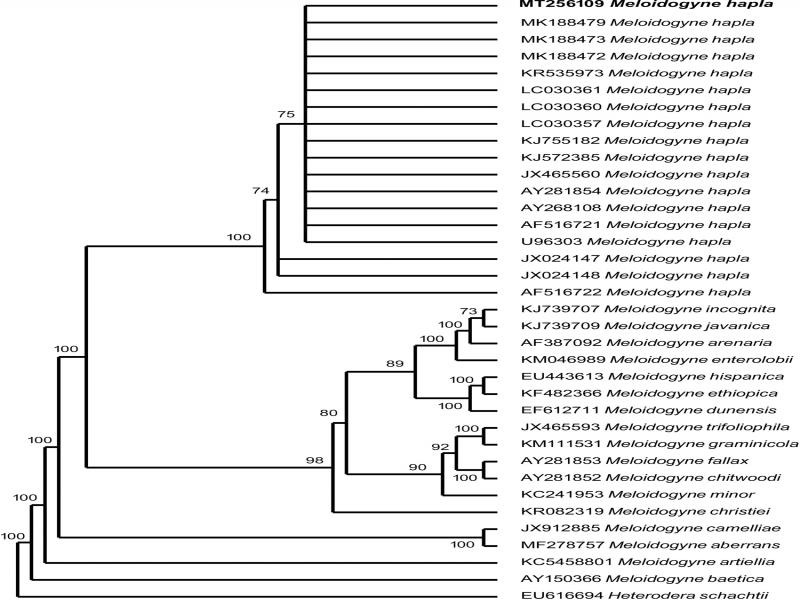
The Bayesian tree inferred from known and newly sequenced *Meloidogyne hapla* from South Africa based on the ITS rDNA region under GTR + I + G model (−lnL = 7,888.3530; *K* = 80; freqA = 0.2366; freqC = 0.2071; freqG = 0.2510; freqT = 0.3053; R(a) [AC] = 1.8343; R(b) [AG] = 2.6987; R(c) [AT] = 3.2232; R(d) [CG] = 1.2677; R(e) [CT] = 3.5360; R(f) [GT] = 1.0000; p-inv = 0.0000; gamma shape = 0.7770).

**Figure 3:**
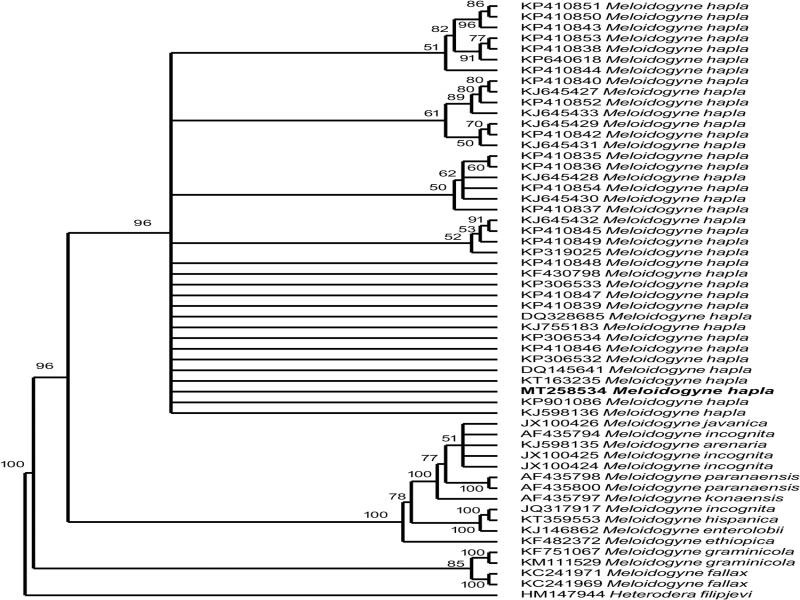
The Bayesian tree inferred from known and newly sequenced *Meloidogyne hapla* from South Africa based on the 28S rDNA region under GTR + I + G model (−lnL = 7,780.8382; *K* = 122; freqA = 0.2627; freqC = 0.2243; freqG = 0.2384; freqT = 0.2747; R(a) [AC] = 0.8857; R(b) [AG] = 1.6067; R(c) [AT] = 1.0059; R(d) [CG] = 0.7613; R(e) [CT] = 2.1749; R(f) [GT] = 1.0000; p-inv = 0.0000; gamma shape = 1.0490).
